# Digital immunohistochemistry wizard: image analysis-assisted stereology tool to produce reference data set for calibration and quality control

**DOI:** 10.1186/1746-1596-9-S1-S8

**Published:** 2014-12-19

**Authors:** Benoît Plancoulaine, Aida Laurinaviciene, Raimundas Meskauskas, Indra Baltrusaityte, Justinas Besusparis, Paulette Herlin, Arvydas Laurinavicius

**Affiliations:** 1 Path-Image/BioTiCla, University of Normandy, Unicaen, Caen, France; 2 Department of Pathology, Forensic Medicine and Pharmacology, Faculty of Medicine, Vilnius University, Vilnius, Lithuania; 3 National Center of Pathology, affiliate of Vilnius University Hospital Santariskiu Clinics, Vilnius, Lithuania

## Abstract

**Background:**

Digital image analysis (DIA) enables better reproducibility of immunohistochemistry (IHC) studies. Nevertheless, accuracy of the DIA methods needs to be ensured, demanding production of reference data sets. We have reported on methodology to calibrate DIA for Ki67 IHC in breast cancer tissue based on reference data obtained by stereology grid count. To produce the reference data more efficiently, we propose digital IHC wizard generating initial cell marks to be verified by experts.

**Methods:**

Digital images of proliferation marker Ki67 IHC from 158 patients (one tissue microarray spot per patient) with an invasive ductal carcinoma of the breast were used. Manual data (mD) were obtained by marking Ki67-positive and negative tumour cells, using a stereological method for 2D object enumeration. DIA was used as an initial step in stereology grid count to generate the digital data (dD) marks by Aperio Genie and Nuclear algorithms. The dD were collected into XML files from the DIA markup images and overlaid on the original spots along with the stereology grid. The expert correction of the dD marks resulted in corrected data (cD). The percentages of Ki67 positive tumour cells per spot in the mD, dD, and cD sets were compared by single linear regression analysis. Efficiency of cD production was estimated based on manual editing effort.

**Results:**

The percentage of Ki67-positive tumor cells was in very good agreement in the mD, dD, and cD sets: regression of cD from dD (R^2^=0.92) reflects the impact of the expert editing the dD as well as accuracy of the DIA used; regression of the cD from the mD (R^2^=0.94) represents the consistency of the DIA-assisted ground truth (cD) with the manual procedure. Nevertheless, the accuracy of detection of individual tumour cells was much lower: in average, 18 and 219 marks per spot were edited due to the Genie and Nuclear algorithm errors, respectively. The DIA-assisted cD production in our experiment saved approximately 2/3 of manual marking.

**Conclusions:**

Digital IHC wizard enabled DIA-assisted stereology to produce reference data in a consistent and efficient way. It can provide quality control measure for appraising accuracy of the DIA steps.

## Background

Digital image analysis (DIA) brings great perspectives to improve reproducibility and capacity of immunohistochemistry (IHC) studies. Nevertheless, the benefits of reproducibility, capacity and clinical utility are not a substitute for accuracy or objectivity of the DIA methods established by comparison to reference data or criterion standard, preferably representing same type of data obtained by direct and more reliable measurement [1,2]. While validation studies in digital IHC commonly relied on pathologists' visual evaluation or biologic ground truth (e.g., HER2 FISH [3]) as a criterion standard, more recently the effort was put into manual counting of cells to obtain the reference data [4,5].

We have recently reported [6] on methodology to validate and calibrate DIA for Ki67 IHC in breast cancer tissue based on reference data obtained by stereology grid count performed on the same images: comparison of the DIA results to the reference data enabled "knowledge-based" fine-tuning of the DIA settings to achieve better accuracy. Although the methodology was successful, its practical application would be time-consuming and dependent on expert human resource to produce the data. In the perspective of multiple IHC markers to be analyzed with variable IHC staining protocols and scanning platforms, the efficiency of continuous quality control and production of the data sets becomes an important prerequisite. This demand has been recognized in broad field of bio-image informatics with the statement that full-scale adoption of automated DIA tools would require efficient production and maintenance of the data sets and provision of integrated editing tools [7]. The amount of manual expert work needed on large images with a huge number of cells is a critical bottle-neck in adoption of the editing-based approaches [8]. In brain tissue analyses, sophisticated approaches have been proposed to decrease the workload by calculating a segmentation confidence score for each cell [9] or identifying potential outliers to prioritize the expert review [10].

Clinical adoption of digital IHC techniques could be synergized by tools enabling efficient production of reference data sets for divergent IHC applications requiring proper quality controls. To the best of our knowledge, editing-based approaches have not been developed in the field of digital IHC. We therefore present a technique (dIHC Wizard) which combines the swiftness of image processing and the unbiased approach of stereology and which aims at alleviating manual workload of the expert in obtaining reference data sets. DIA is used as an initial step in stereology grid count procedure to reduce the need for manual work. The DIA-generated data (dD) are then edited by an expert to produce the corrected data (cD) which can be regarded as quality-assured data and be used as the standard criterion for further DIA calibrations.

## Methods

**Figure 1:**
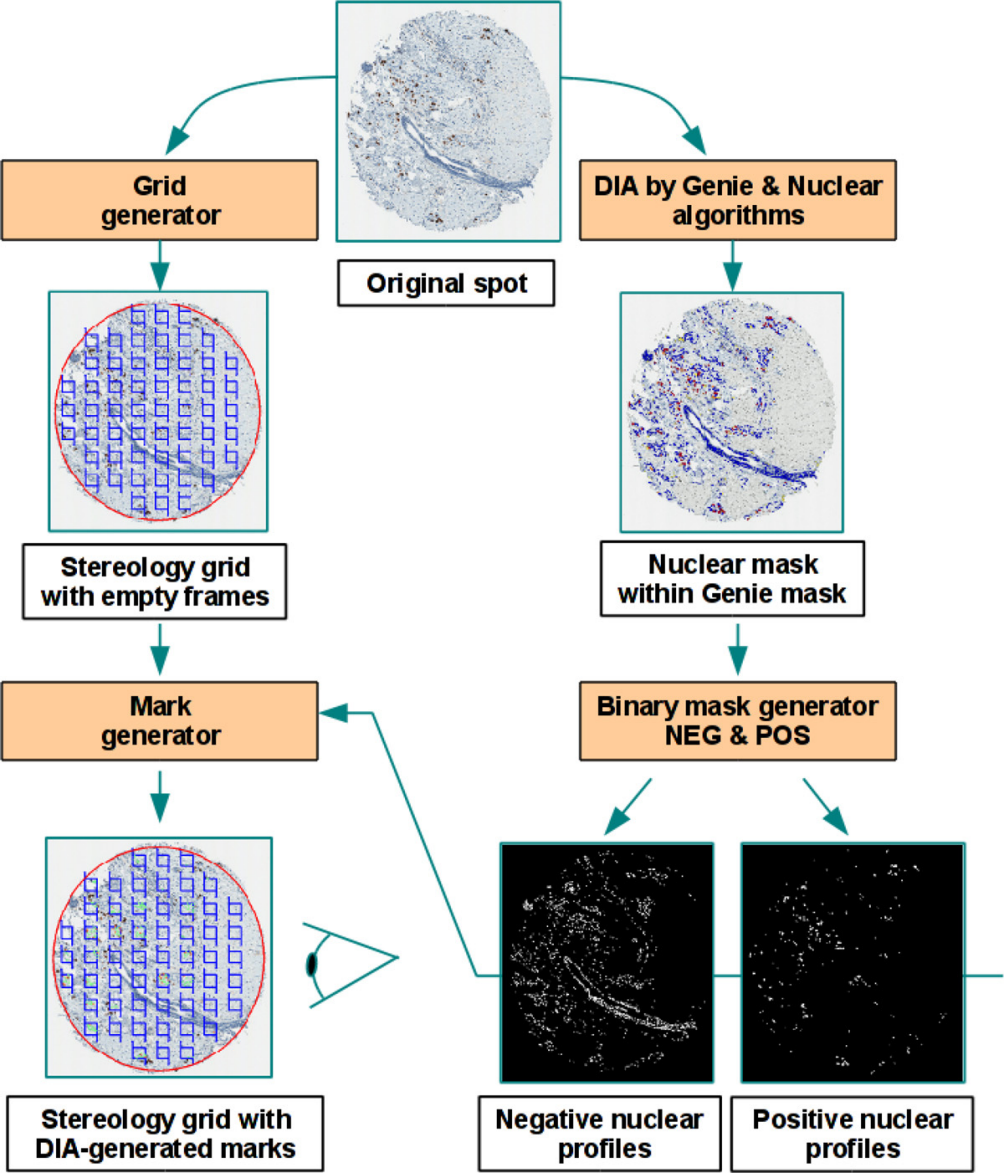
**dIHC Wizard workflow, the process of the XML tags (Grid and Mark generator) at left and the process of the image analysis (Genie and Nuclear algorithms and binary mask generator) at right**.

**Figure 2:**
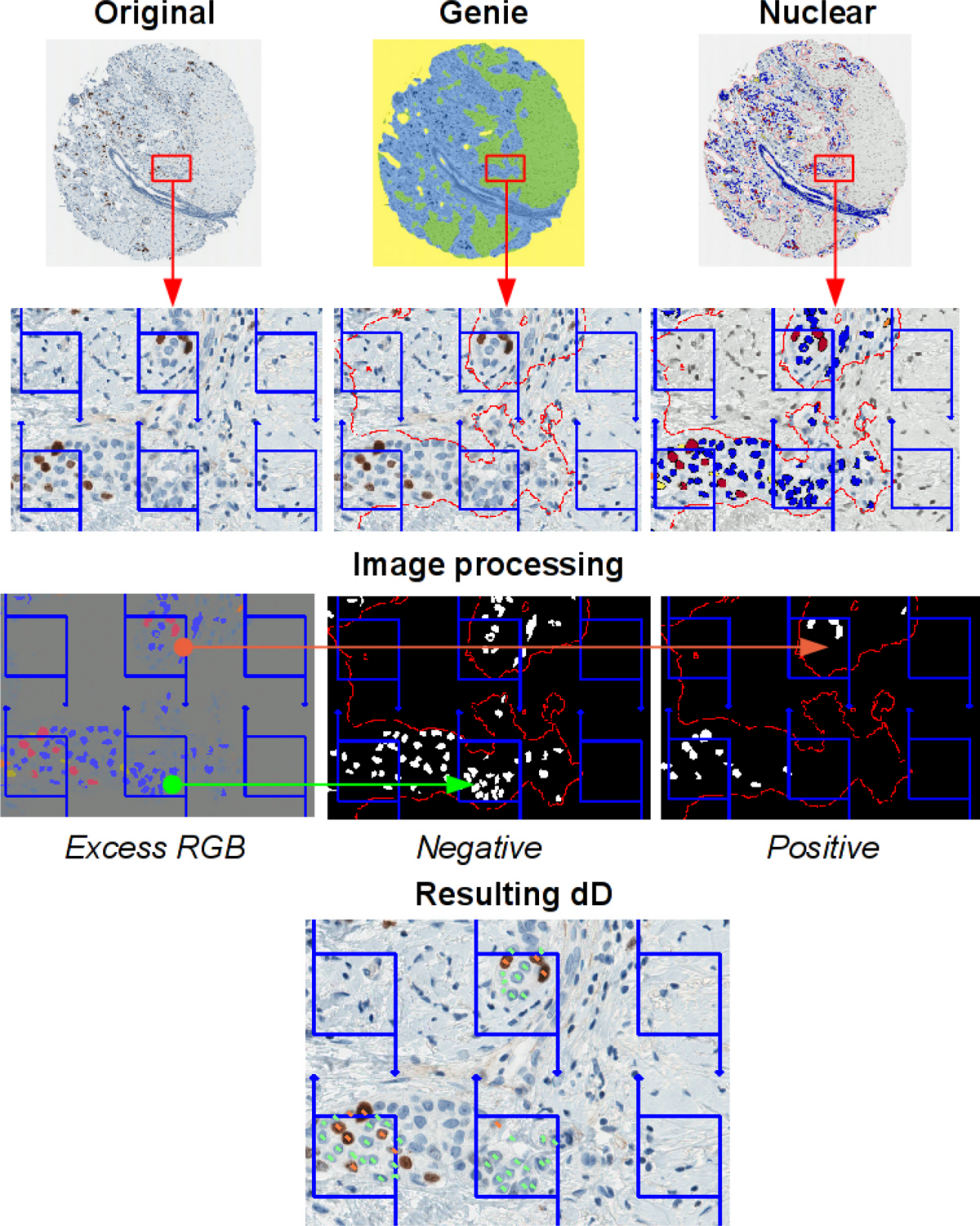
**Building of dD marks**: Original (initial spot image), Genie (spot image processed by the Genie software), Nuclear (spot image processed by the Nuclear software in the Genie mask), Excess RGB (computation of an excess red, green and blue image from the Nuclear resulting image), Negative and Positive (binary masks resulting from Excess RGB image thresholding), Resulting dD (test grid of frames with marks of the positive, in orange, and negative, in green, nuclear sections).

**Figure 3:**
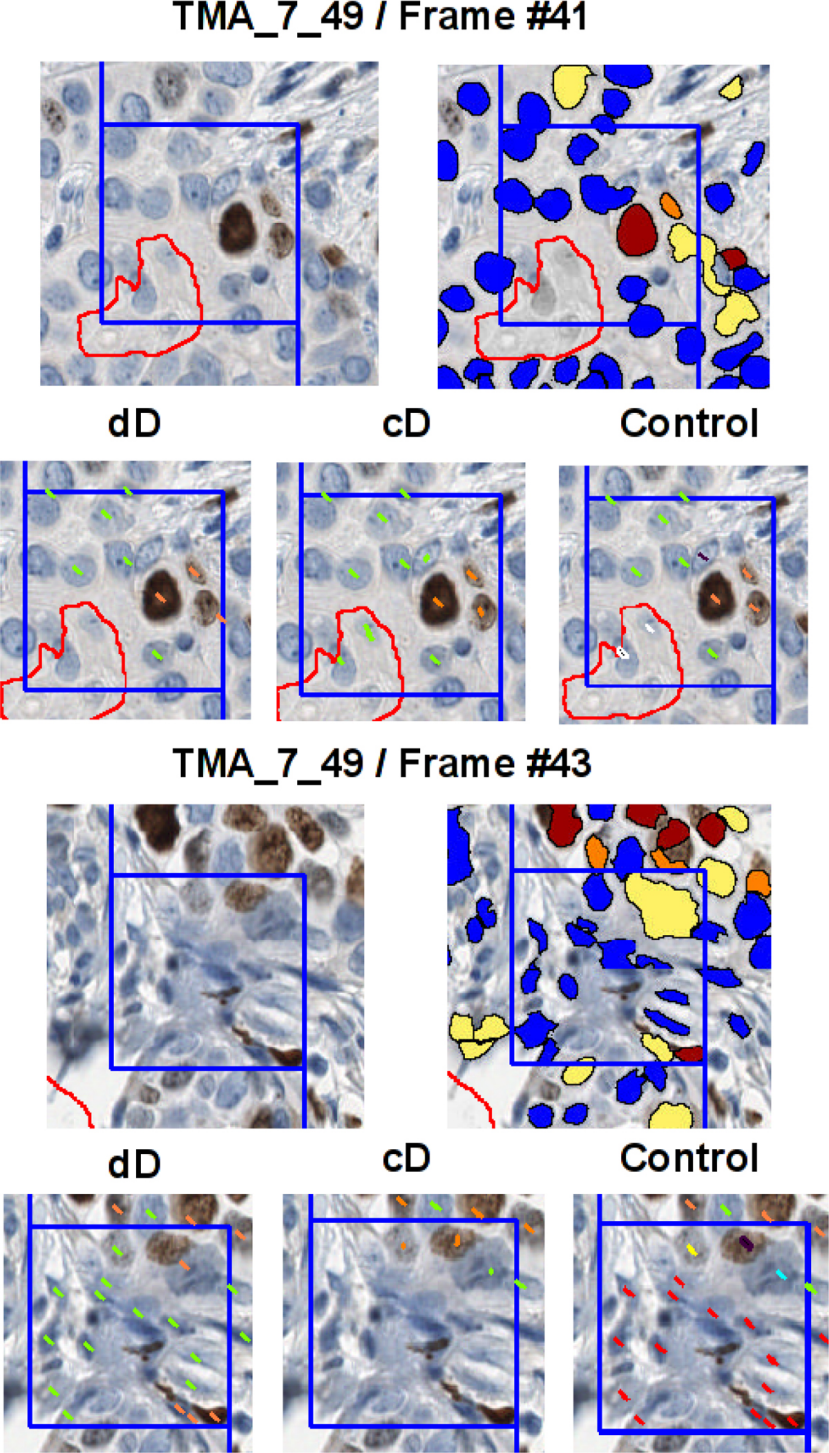
**Comparison of dD and cD, visual illustration for a spot image (TMA_7_49) and two frames (Frame #41 and Frame #43)**: red outline (contour of Genie mask), blue outline (contour of the frame with forbidden line), orange marks (true positive nuclei), yellow marks (false positives), green marks (true negatives), light blue marks (false negatives), red marks (nuclei falsely detected by Genie or Nuclear software), white marks (epithelial cell nuclei undetected by Genie software), black marks (epithelial cell nuclei undetected by Nuclear software).

Digital images of Ki67 IHC from 158 female patients (one millimeter-diameter tissue microarray (TMA) spot per patient) with an invasive ductal carcinoma of the breast were used for the study. The study was approved by the Lithuanian Bioethics Committee. The patients' consent to participate in the study was obtained. The TMA were constructed, stained, and scanned as previously described [11]. Manual data (mD) were obtained in our previous study [6] by marking Ki67-positive and negative tumour cell profiles, using a stereological method for 2D object enumeration [12] implemented in the Stereology module (ADCIS, France) with a test grid of systematically sampled frames overlaid on a spot image in ImageScope (Aperio Technologies, USA). Automatic image processing was performed using the combination of the Genie and Nuclear algorithms from Aperio Technologies (Vista USA) calibrated in the previous study [6]. The Genie software is used to isolate the epithelial compartment of the breast carcinoma and discard stromal and inflammatory components. Nuclear algorithm allows the refined segmentation of individual nuclei of cancer cell profiles and the color characterization of immunostained and counterstained nuclei. The dIHC Wizard workflow is presented in the Figure 1. The dD marks were produced from the multicolor mask image resulting from the automatic analysis. For this purpose, an Excess Red Green Blue image was computed (2R-G-B, 2G-R-B and 2B-R-G) and fixed thresholds applied to isolate the automatically labeled immunostained and counterstained epithelial nuclear profiles (Figure 2). The dD marks were collected from the centroid of the labeled nuclear profiles and stored into an XML file which was then overlaid on the original spot images to generate relevant XML tags along with the stereology grid (Figure 2). This allowed the expert to edit, by manual adding or deleting, the dD marks, as needed to produce the cD. The mD and cD were produced by the same experts. A new file containing XML tags representing the cD was generated and compared to the original file (dD) to retrieve data for statistical analysis (Figure 3). The percentage of Ki67 positive tumour cell profiles per spot (Ki67%) was used to compare the data sets. True and false positive and negative Ki67 tumor cell rates as well as lost or false tumor cells were established for each spot. The mD, dD, and cD sets were compared by single linear regression analysis. Agreement between individual measurements was also estimated and visualized by Bland and Altman plots [13]. Furthermore, the undetected tumor cells were subcategorized to "By the Genie" and "By the Nuclear" algorithms based on reference of their coordinates to the original DIA markup images. Falsely detected tumor cells were due to the Genie or the Nuclear components. This allowed comparing the impact of the Genie and Nuclear algorithms on dD error rate providing insights on relative accuracy of each DIA component. Efficiency of cD production was estimated based on expert editing effort required. Statistical significance level was set at p < 0.05.

## Results and discussion

**Figure 4:**
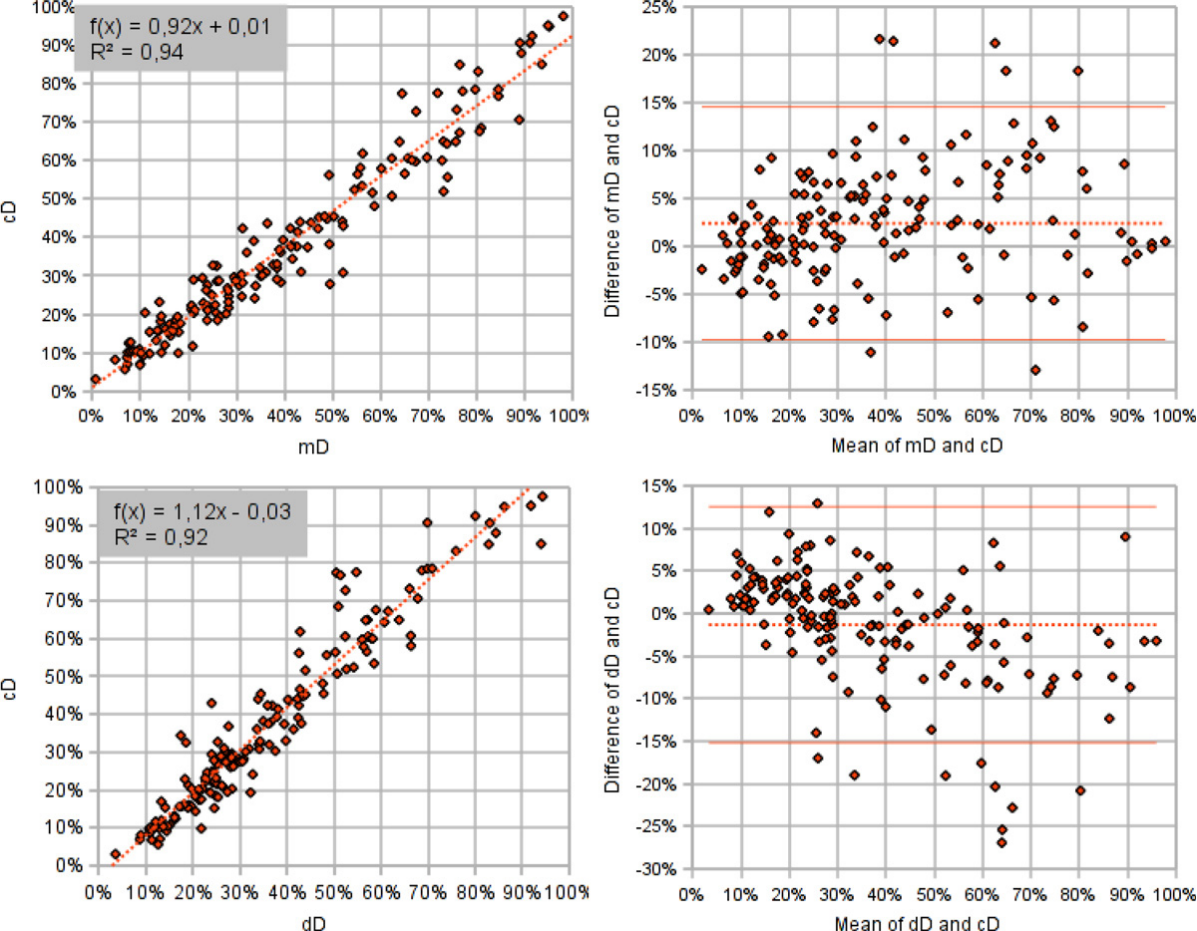
**Upwards, comparison between mD and cD by single linear regression analysis at left and Bland Altman analysis at right; downwards, comparison between dD and cD by single linear regression analysis at left and Bland Altman analysis at right**.

The percentage of Ki67-positive tumor cells in each spot image (Ki67%) was in a very good agreement in the mD, dD, and cD sets when compared by single linear regression analysis (Figure 4). Regression of the cD from the dD (R^2 ^= 0.92) reflects the impact of the expert editing the dD as well as accuracy of the DIA used to produce the dD. Regression of the cD from the mD (R^2 ^= 0.94) reflects the consistency of the DIA-assisted standard criterion (cD) production with the manually obtained standard criterion. Slight bias in the opposite directions can be noted in both comparisons.

**Figure 5:**
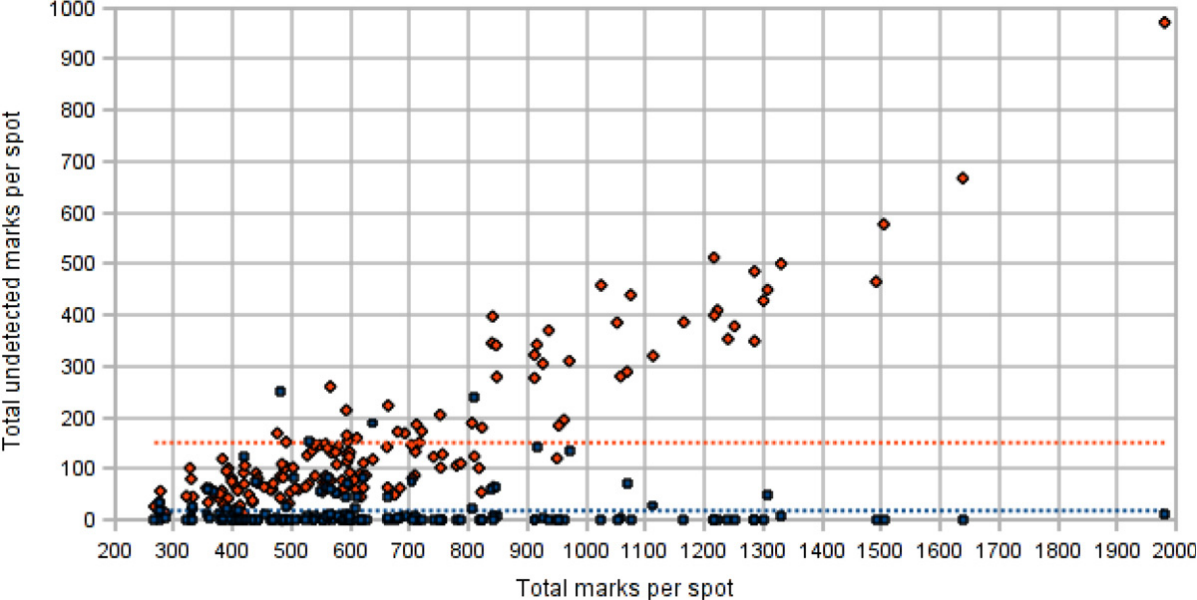
**Numbers of tumor cell nuclei undetected by the Genie (blue dots) and the Nuclear (orange dots) algorithms**.

**Figure 6:**
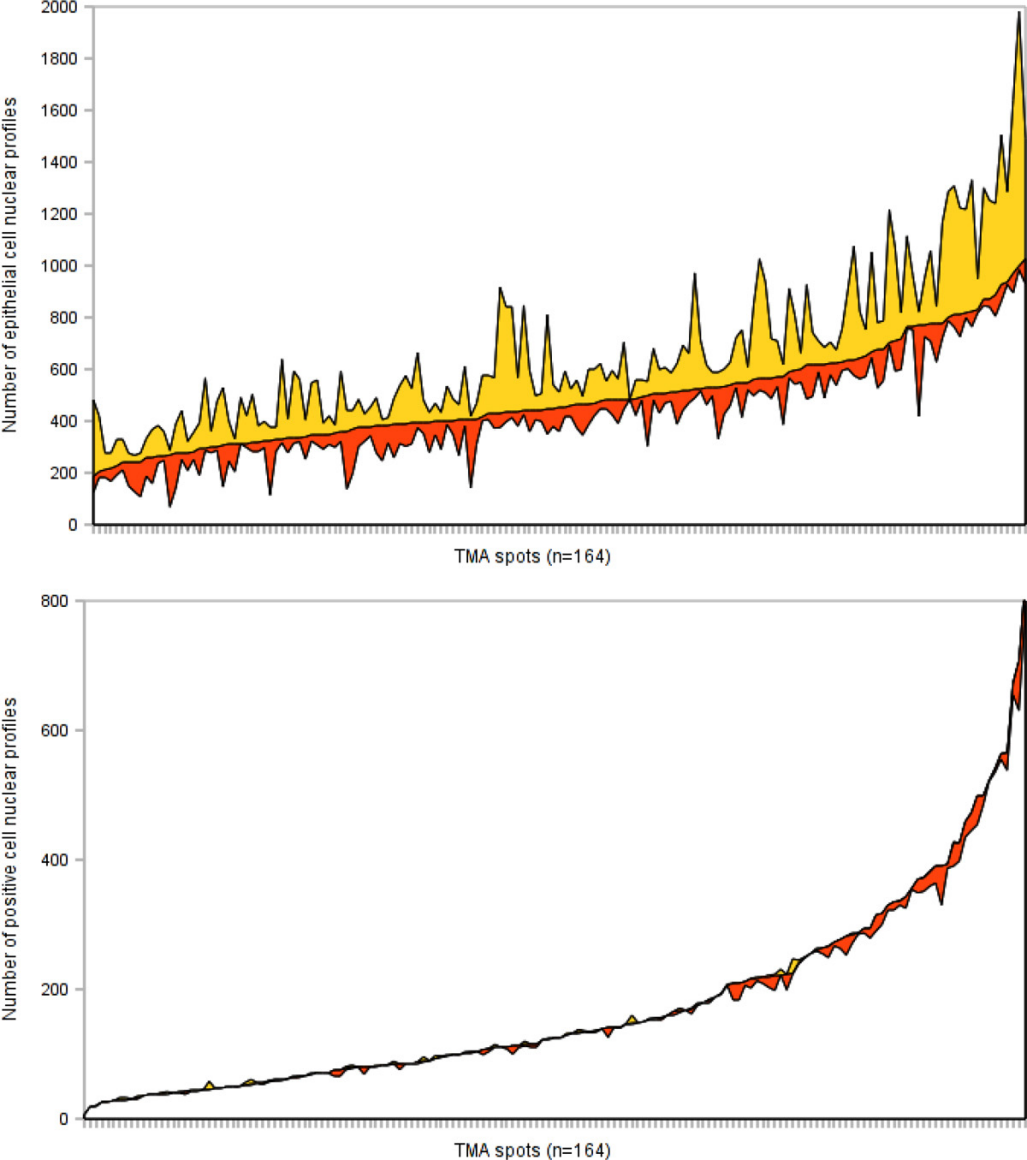
**Upwards, the efforts of the experts for correcting undetected (yellow color area) and falsely detected (orange color area) epithelial cell nuclei and downwards, the efforts for correcting falsely labeled (yellow color area) and unlabeled nuclei (orange color area) for the whole TMA spot series, presented on the x axis and sorted ascending by the value of the Y axis**.

Besides the comparison of the summarized TMA spot indicators in the data sets, our method provides detailed information on accuracy of detection and the IHC-positivity interpretation by DIA of individual cells. Despite the very good agreement of the Ki67% per spot between the data sets, the accuracy of detection of individual tumor cells was much lower. Based on the expert corrections made on the dD to produce the cD, in average 18 and 219 marks per spot were edited due to the Genie and Nuclear algorithm errors, respectively (Figure 5); the mean of marks per spot was 663. In all 158 TMA spots, a total of 105,486 nuclei have been identified in the cD while 39,710 (37.6%) expert corrections have been made on the dD, including 2,941 (2.8%) and 24,727 (23.4%) edits to correct the Genie and Nuclear under-detection of epithelial nuclear profiles, respectively, and 10,793 (10.2%) over-detection of epithelial nuclear profiles by Genie or Nuclear. As described in the Methods section, the Nuclear component of the DIA was applied only on the epithelial mask already detected by the Genie component, therefore, the performance indicators of the Nuclear detection component are "Genie-dependent". When nucleus is properly identified, the IHC staining interpretation (Ki67-positive *versus *negative) by the Nuclear algorithm can be regarded as excellent: overall, only 1,035 (1%) false positive and 214 (0.2%) false negative tumor nuclei were corrected by the experts (Figure 6).

Importantly, our data highlight the impact of the accuracy definition and the DIA validation results: it was perfect with regard to the Ki67% result per image which could be interpreted as sufficient for clinical use. However, the accuracy of the individual tumour cell detection was less satisfactory and may be taken as a warning sign on the road of developing robust automated DIA tools. Perhaps, different applications may require more conservative accuracy definitions and validation procedures for specific DIA tasks.

Furthermore, our approach provided a benefit of "decomposing" the accuracy of 3 DIA components used by one expert editing procedure. Importantly, the Genie component outperformed the Nuclear algorithm in terms of tumor cell detection in our experiment. We are not aware of published data on the relative impact on DIA accuracy caused by automated tumour (epithelial) tissue and tumour cell identification components. While the issue of automatic detection and segmentation of cell nuclei in histopathology images is well-addressed, accuracy of tumour tissue detection would require targeted studies. Lastly, discrimination between the Ki67 IHC staining result (positive *versus *negative) by the DIA was excellent in our study.

We therefore suggest that even if the accuracy of the Ki67% (image-based estimate) on the whole spot series was very good, more conservative cell-based validation approach could uncover the "functional anatomy" of the DIA tools and point further DIA improvement efforts in the right direction to achieve most robust DIA processes and results.

The accuracy of the DIA tool used to produce the dD set determines the efficiency of the DIA-assisted cD production. In our case, the approach saved approximately 2/3 of manual editing, however, the effort to review the images remained the same. The efficiency can be further increased by improving ergonomics of the stereology tool and employing a better calibrated DIA-assistance. In our experiment, we applied systematic random sampling by stereology grid; it is a simple method to control the amount of manual work, however, it is subject to variable amount of tumour tissue and cellularity in the images. This disadvantage can be compensated by automated resizing of the stereology grid based on the results of the DIA to produce the dD set in order to get optimal number of cells to be reviewed per image.

Last but not least, the mD and/or cD sets are quality-assured and contain information on exact location of the cells in the image, therefore, can be utilized as standard criterion templates to validate, calibrate, and train DIA tools. On a more global perspective, the reference data libraries may serve as benchmark datasets for automated DIA - the demand well-recognized and addressed in computational neuroscience and bioimage informatics, in general, with tasks of much higher complexity than the digital IHC [14,15].

## Conclusions

We propose the dIHC Wizard to enable image analysis-assisted stereology approach to produce reference data in a more efficient way. In our experiment, it reduced the expert manual work up to 3 times. Importantly, the tool can also support DIA quality control and improvement efforts since it provides detailed information on the accuracy of the DIA components applied - tumour tissue and tumour cell detection as well as interpretation of the Ki67 IHC positivity in the detected cells.

## List of abbreviations

DIA: digital image analysis; IHC: immunohistochemistry; Ki67%: the percentage of Ki67-positive tumor cells in each spot image; cD: corrected data; dD: digital data; mD: manual data; TMA: tissue microarray.

## Competing interests

The authors declare that they have no competing interests.

## Authors' contributions

BP designed and produced the software for the study, drafted the manuscript, performed statistical analyses. AL and PH drafted essential parts of the manuscript, participated in statistical analyses. AiL designed and carried out the digital image analyses, supervised the expert review procedures, edited the manuscript. AiL, IB, JB, RM performed the expert review. All authors participated in conception and design of the study, reviewing the analysis results, critically revised and approved the final manuscript.

## Authors' information

None.
